# Continuous oral chloroquine as a novel route for *Plasmodium *prophylaxis and cure in experimental murine models

**DOI:** 10.1186/1756-0500-4-262

**Published:** 2011-07-28

**Authors:** Matthew D Lewis, Johannes Pfeil, Ann-Kristin Mueller

**Affiliations:** 1 Department of Infectious Diseases, Parasitology Unit, Heidelberg University School of Medicine, Im Neuenheimer Feld 324, D 69120 Heidelberg, Germany; 2 Angelika-Lautenschläger-Kinderklinik, Zentrum für Kinder-und Jugendmedizin, Heidelberg University School of Medicine, Im Neuenheimer Feld 430, D 69120 Heidelberg, Germany

## Abstract

**Background:**

Chloroquine (CQ) is utilized as both cure and prophylaxis to *Plasmodium *infection. In animal studies, CQ administration to experimental animals is via intraperitoneal (i.p.) injection of a single dose that varies from daily to several times per week. Such daily administration can be distressing to the animals and provoke aggressive behaviors that may affect the immune responses of the animal and interfere with data read-outs.

**Findings:**

We describe a novel, viable and efficacious prophylactic and curative administration route whereby chloroquine is continuously supplied in the drinking water to experimental animals. The prophylactic effect is robust and the curative effect against patent blood stage infection comparable to the traditional route of i.p. administration. Continuous drinking water administration may decrease animal stress responses and thus improve the reliability of experimental data.

## Findings

Chloroquine (CQ) is a classic 4-aminoquinoline that displays rapid schizonticidal activity against the blood stages of many *Plasmodium *species. Accumulating at high concentrations within the blood-stage digestive vacuole [1] it forms non-covalent complexes with heme [2], interferes with heme sequestration to the less toxic product hemozoin and poisons the parasite via the accretion of such drug-heme complexes [3]. Discovered in 1934 and introduced into clinical practise in 1947 as both treatment and primary prophylaxis to malaria disease, it remains the first drug of choice in most sub-Saharan African countries despite the emergence of resistant strains in the late 1950s [4] harboring the *Plasmodium falciparum *chloroquine resistance transporter (*Pf*CRT) gene [5].

In the laboratory, CQ remains a useful tool, particularly when studying the immunology of murine *Plasmodium *infection. Since the onset of clinical symptoms are concomitant with the infection of red cells, CQ is often administered as prophylaxis so that the parasite infection manifests within the liver stage but does not develop to blood-stage infection and pathology [6,7]. The administration of CQ to experimental animals in such studies is in nearly all occasions via intraperitoneal (i.p.) injection of a single dose that varies from daily to several times per week. In large studies, the daily administration of i.p. injections to large-scale-cohorts of mice can be time-consuming, distressing to the animals and provoke aggressive behaviors that may affect the immune responses of the animal and thus interfere with data read-outs [8,9]. Thus we explored the oral administration route as a prophylactic and curative means of controlling and eliminating *Plasmodium berghei *ANKA infection in C57BL/6 mice via continuous (and unstopped) administration in the drinking water as a convenient, low-interference administration method.

The water uptake of mice is between 4 and 7 ml per day depending upon air temperature and humidity [10]. Based on this volume we calculated a concentration of CQ salt of 0.288 mg/ml dissolved in normal tap water to deliver a daily oral dose of 1.15 mg to 2.02 mg. This is broadly comparable to the concentrations delivered i.p., which themselves vary from study to study [6,7]. A small amount of glucose powder (approximately 15 g/l) was dissolved in the CQ solution (hereafter referred to as CQ-DW) to make it more palatable to the animals and encourage consumption. Once dissolved, the solution was administered in tinted light-resistant bottles and refreshed weekly. No other liquid source was available.

**Figure 1:**
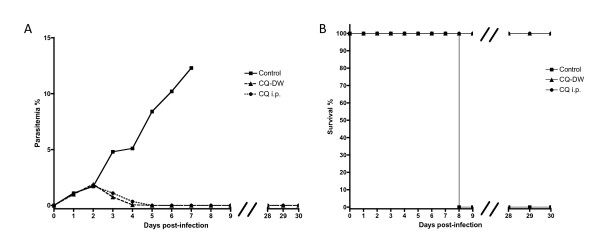
**CQ-DW has a curative effect similar to i.p. administration in experimental animals**. C57BL/6 mice (n = 20) were infected with 10^6 ^parasitized erythrocytes by i.v. injection at day 0. At day 2 post-infection, and thereafter daily, mice were administered chloroquine drinking water preparation (CQ-DW) or i.p. injection of chloroquine (CQ i.p.). Parasitemia percentage was calculated by Giemsa stain of blood smears (A) and animal survival was noted (B).

To test the curative effect of CQ-DW against a pre-established infection versus the traditional route of i.p. administration, animals (n = 10) were injected with 10^6 ^parasitized *P. berghei *ANKA erythrocytes in the tail vein i.v.. Two days later these animals had parasitemias of 1.5-2%. CQ was administered by CQ-DW (n = 5) and by i.p. injection (n = 5) of 100 μl solution dissolved in PBS at a concentration of 8 mg/ml. In both groups, an immediate and clear inhibitory effect upon the parasite infection could be observed the next day, with blood stages undetectable by microscopic analysis day 3 post-CQ-DW treatment. CQ-DW was maintained until day 7 post-treatment whereupon it was replaced with normal water. Mice were observed for a further 30 days and no parasites were detectable by microscopic examination of Giemsa-stained blood smears (Figure 1a). Control mice succumbed to experimental cerebral malaria at day 8 (Figure 1b).

**Figure 2:**
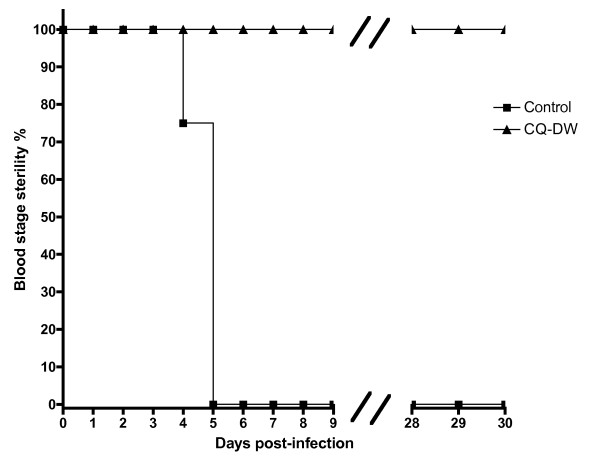
**CQ-DW has a prophylactic effect in experimental animals**. C57BL/6 mice (n = 25) were infected with 10,000 sporozoites by i.v. injection at day 0. Mice were immediately administered chloroquine drinking water preparation (CQ-DW). "Blood stage sterility" was assessed by Giemsa stain of blood smears, with non-sterility diagnosed upon the irrefutable detection of two parasites per slide.

To test the prophylactic efficacy of CQ-DW, a separate group of animals (n = 25) was infected by intravenous (i.v.) injection of 10,000 purified *Plasmodium berghei *ANKA sporozoites in the tail vein and then normal drinking water was replaced with CQ-DW in a test group (n = 20) and not in the control group (n = 5). Mice were monitored until day 30 post-infection by daily analysis of blood smears stained with Giemsa. No parasites were observed in the test group during this period and control mice became patent at days 4 - 5 (Figure 2). Upon replacing CQ-DW with normal water, test mice were divided into a further two groups. One test group (n = 10) was challenged intravenously with 10^6 ^parasitized erythrocytes some five days after CQ-DW replacement and a second control group (n = 10) was monitored. Blood infection remained negative in the control group during a further observation period of 30 days. In the test group, mice were found to be susceptible to re-infection, with parasites observed by Giemsa stain 24 hours post-challenge (data not shown).

Thus CQ-DW offers a convenient and robust method for chloroquine treatment and prophylaxis in experimental murine *Plasmodium *infection protocols. Over the course of the experiments we did not observe any adverse effects of continuous CQ treatment on the experimental animals. Of additional note is that, unlike pyrimethamine-drinking water preparations that produce a rancid odor and is seemingly associated with a reduction in consumption volumes to minimal levels (unpublished own observations), animals are apparently satisfied to drink CQ-DW, as we observed no change in drinking patterns or habits between normal and CQ-DW administration. Also, it is worth noting that there is a "point of no return" in CQ-DW administration, since in animals with advanced blood-stage infections and such debilitating malarial symptoms that they are incapable of liquid consumption, or show significant lethargy or coma, the nature of "self-administration" of CQ-DW is nullified. Additionally, one inherent limitation to this means of administration is that dosage will vary between individual animals depending upon volume consumed. However, for the purpose of maintaining prophylaxis or curing patent infection, despite potential minor variability in exact dosage, our work demonstrates that CQ-DW administration is efficacious, with robust prophylactic protection and clearance of pre-existing patent parasitemias comparable to the established route of i.p. administration. When considering the applicability of such a method to humans in endemic areas we must be highly cautious, due to considerations such as parasite resistance and the toxic effects associated with CQ treatment [11,12]. However, despite this, it is conceivable that the administration of individual doses of chloroquine via a dissolving seltzer tablet in water may be a favourable dispensing method over tablets, much like the syrup preparations that are commonly prescribed for infants and young children [13]. Moreover, in the laboratory mouse, it is an efficacious and viable experimental protocol that saves time for the researcher and improves animal welfare standards, perhaps in turn improving the reliability of experimental data.

## List of abbreviations used

CQ: Chloroquine salt; CQ-DW: Drinking water supplemented with chloroquine.

## Ethics Statement

All animal experiments were performed according to FELASA category B and GV-SOLAS standard guidelines. Animal experiments were approved by German authorities (Regierungspräsidium Karlsruhe, Germany), § 8 Abs. 1 Tierschutzgesetz (TierSchG).

## Competing interests

The authors declare that they have no competing interests.

## Authors' contributions

Experiments conceived by MDL, JP and AKM. The manuscript was written by MDL and AKM. The authors read and approved the manuscript.
